# Heat Tolerance Differences Between Hu Sheep and Hu Crossbred Sheep in Microbial Community Structure and Metabolism

**DOI:** 10.3390/metabo15010040

**Published:** 2025-01-10

**Authors:** Jing-Da Yuan, Li-Wei Wang, Shao-Yin Fu, Ri-Ge-Li-Tu E, Xiao-Qi Ren, Hua Sun, Fang Liu, Biao Wang, Jiang-Hong An, Meng-Ran Zhao, Jiang-Feng He, Xiao-Long He

**Affiliations:** 1 Inner Mongolia Academy of Agricultural & Animal Husbandry Sciences, Hohhot 010031, China; yuanjingda0825@163.com (J.-D.Y.); 18747977760@163.com (L.-W.W.); ffsy@imaaahs.ac.cn (S.-Y.F.); 15144714461@163.com (R.-G.-L.-T.E.); 18747599252@139.com (X.-Q.R.); sunhua564116908@163.com (H.S.); liuf185@163.com (F.L.); wb@imaaahs.ac.cn (B.W.); an15354856117@sina.com (J.-H.A.); burutiaowuyjd@163.com (M.-R.Z.); 2 College of Animal Science and Technology, Inner Mongolia Minzu University, Tongliao 028000, China

**Keywords:** heat stress, meat sheep, thermotolerance, metabolome, gut microbiota

## Abstract

Background: The frequent occurrence of extreme temperature events causes significant economic losses to the livestock industry. Therefore, delving into the differences in the physiological and molecular mechanisms of heat stress across different sheep breeds is crucial for developing effective management and breeding strategies. Methods: This study explores the differences in heat tolerance mechanisms between Hu sheep and Xinggao sheep by comparing their growth performance under normal and heat stress conditions, as well as examining the differences in physiological, biochemical, and antioxidant indicators related to heat tolerance, serum metabolomics, and gut microbiomics in a heat stress environment. Results: The results indicate that with changes in the temperature–humidity index (THI), Hu sheep exhibit superior stability in respiratory rate (RR) and rectal temperature (RT) fluctuations compared to Xinggao sheep. In terms of biochemical indicators and antioxidant capacity, the levels of creatinine (Cr) and superoxide dismutase (SOD) in Hu sheep serum are significantly higher than those in Xinggao sheep. In comparison, alkaline phosphatase (ALP) and malondialdehyde (MDA) levels are significantly lower. Metabolomic results showed that, compared to Hu sheep, Xinggao sheep exhibited higher cortisol (COR) and dopamine (DA) levels under heat stress conditions, a stronger lipid mobilization capacity, and elevated levels of tricarboxylic acid (TCA) cycle-related metabolites. Furthermore, gut microbiome analysis results indicate that Hu sheep demonstrate stronger cellulose degradation capabilities, as evidenced by significantly higher abundances of microorganisms such as *Ruminococcus*, *Fibrobacter*, and *Bacteroidales_RF16_group*, compared to Xinggao sheep. Conclusions: In summary, Hu sheep exhibit stronger heat tolerance compared to Xinggao sheep. These findings provide an important theoretical basis for the breeding and selection of heat-tolerant meat sheep varieties and offer strong support for the region’s livestock industry in addressing the challenges posed by global warming.

## 1. Introduction

The latest research by the Intergovernmental Panel on Climate Change (IPCC) shows that the rate of global warming in recent years has far exceeded expectations. The ongoing extremely high temperatures have not only impacted animal welfare but have also caused significant economic losses to the livestock industry [1]. According to statistics, the economic losses in the livestock industry due to heat stress in the United States alone exceed 1.2 billion dollars each year [2]. Consequently, mitigating the adverse effects of extreme heat stress on sheep farming has become a critical focus of animal husbandry research. In addition to the inherent challenges posed by climate change, the increase in stocking density and environmental humidity resulting from intensive farming practices, driven by policies aimed at reducing grazing pressure and increasing domestic demand, has exacerbated the impact of heat stress on confined sheep. These changes highlight the urgent need for breeds with enhanced heat tolerance.

The Hu sheep, native to the humid and hot regions of southern China, is a breed used for lambskin; however, in recent years, with the decline of the lambskin market and the growth of the meat sheep industry, as well as Hu sheep’s high reproductive capacity and suitability for intensive farming (strong heat tolerance and rapid growth rate), it has been extensively introduced into concentrated areas of meat sheep production in northern China for meat production [3]. Simultaneously, this industrial shift has prompted researchers and breeders to increasingly focus on the meat production performance and reproductive capabilities of sheep. Against this backdrop, a new breed of Xinggao sheep has been developed, which is a hybrid created by crossing Hu sheep as the maternal line with Small Tail Han sheep and East Friesian sheep as the paternal lines. Although Xinggao sheep exhibit more outstanding production performance, this may indicate a higher metabolic heat production level. On the other hand, recent climate studies show that northern China is more sensitive to global warming, with a significantly higher rate of temperature increase compared to other regions [1]. However, although these two breeds are widely raised in northern China, their heat tolerance in this region has not yet been studied.

In the context of rapid developments in molecular biology and the increasing costs of animal management, screening heat-resistant breeds has become one of the effective strategies to alleviate heat stress. Liao et al. [4] found through comparative studies on the heat resistance of three beef cattle breeds that the Xuanhan Yellow Cattle adapts to humid and hot environments through a more robust antioxidant system and higher glycolytic activity. Gu et al. [5] indicated that water buffalo exhibit stronger heat resistance than Holstein cattle, a trait closely related to their adaptive responses in branched-chain amino acids, ketogenic amino acids, and carbohydrate metabolism. These studies suggest that different breeds exhibit distinct metabolic regulatory mechanisms in response to heat stress, affecting their heat resistance. The gut microbiome is regarded as the “second genome” of the host and plays a crucial role in regulating the host’s metabolic state. Furthermore, the quantity, composition, and function of the microbiome have a potential causal relationship with the host’s heat resistance [6]. Wang et al. [7] reported that cows with high heat tolerance have higher abundances of the *Succiniclasticum* and *Rikenellaceae_RC9_gut_group* microbiota in their rumens. These microbiota promote propionate production and inhibit methane emissions, which may be closely linked to heat tolerance. Yadav et al. [8] additionally observed that buffalo cows adapt to heat stress environments by modulating the abundance of the phyla *Firmicutes*, *Proteobacteria*, and *Planctomycetes* in their rumens. Currently, research on the heat tolerance of sheep is mainly limited to single omics or biochemical indicators, while systematic exploration of the relationship between metabolites and gut microbiota is still insufficient. Therefore, this study aims to use physiological and biochemical indicators as phenotypes and to deeply reveal the regulatory network between host heat tolerance, metabolites, and gut microbiota through metabolomics and microbiomics techniques. Through this multidimensional analytical approach, we aim to identify key biomarkers related to heat tolerance, thereby comprehensively assessing the differences in heat tolerance between Hu sheep and Xinggao sheep and revealing the molecular mechanisms of their heat tolerance. This research will not only provide scientific evidence for the selection and breeding of sheep breeds better adapted to local climatic conditions but also lay the foundation for promoting the sustainable development of the local meat sheep industry.

## 2. Materials and Methods

### 2.1. Animals and Sample Collection

The animal experiments conducted in this study received approval from the Animal Care and Use Committee at the Inner Mongolia Academy of Agricultural and Animal Husbandry Sciences. A total of twenty 6-month-old ewes, all with similar physiological statuses, were selected for this study, comprising ten Xinggao sheep (XL) and ten Hu sheep (HL). Each group of sheep shared a similar genetic background and was raised in intensive sheep sheds, where they remained in close proximity within the same shed throughout the summer. The sheep were provided with an identical diet twice daily, at 9 am and 5 pm, and had free access to drinking water.

The collection of fecal and blood samples was conducted after a continuous high-temperature period of fifteen days (from July 13 to July 27). Blood samples were collected from the jugular vein of the sheep at noon on July 28 (12:00–14:00). After standing for 20 min, the plasma was centrifuged for 10 min at 2000 rpm to separate the serum samples. Simultaneously, fecal samples were collected by gently squeezing the perianal area of the sheep to obtain fresh feces, which were immediately placed in sterile cryovials. The serum and fecal samples were immediately snap-frozen in liquid nitrogen and then transferred to a −80 °C freezer for storage. The serum samples were used for subsequent biochemical indicators, antioxidant indicators, and serum metabolomics analysis, while the fecal samples were utilized for gut microbiome studies. Additionally, we monitored the Temperature–Humidity Index (THI) and measured the fasting body weight of the sheep using an electronic scale on the mornings of four specific time points: during periods above the thermal neutral zone (July 13 and August 12) and within the thermal neutral zone (September 1 and September 30). These data were used to calculate the daily weight gain of the sheep.

### 2.2. Measurement of Heat Tolerance-Related Environmental and Physiological Indicators

THI values were recorded for the fifteen days (from July 13 to July 27) preceding sampling to illustrate environmental trends. Environmental temperature and relative humidity were recorded hourly using automatic temperature and humidity recorders (accuracy = ±0.5 °C and ±5% relative humidity; resolution = 0.1 °C and 0.1% relative humidity; Elitech Inc., Xuzhou, China). The thermometer was positioned 1.5 m above the floor of the sheep shed. The THI was calculated using the formula proposed by Shi et al. [9], expressed as THI = T − [(0.31 − 0.31 RH) (T − 14.4)], where T denotes ambient temperature (°C) and RH represents relative humidity percentage/100. According to this formula, a THI value of 22.2 or below indicates no heat stress, while a THI of 22.2 or above indicates that sheep are in a state of heat stress [9].

The measurements of respiratory rate and rectal temperature were taken on the mornings (06:00–07:00), afternoons (14:00–15:00), and evenings (19:00–20:00) of the first three days before sample collection (July 25 to July 27). Rectal temperature (RT) was measured with an electronic thermometer (accuracy = ±0.1 °C, measurement range 32.00–42.99 °C; Youmu Inc., Zhengzhou, China). Additionally, a trained observer recorded the respiratory rate (RR) by observing flank movements per minute. According to Benezra’s method, the heat tolerance index (HT) of sheep is calculated using the following formula: HT = (RT/average rectal temperature of the corresponding breed at normal temperature) + (RR/average respiratory rate of the corresponding breed at normal temperature). For the XL group, HT = (RT/39.20) + (RR/30.00); for the HL group, HT = (RT/39.15) + (RR/29.20). A lower HT value indicates higher heat tolerance in sheep [10].

### 2.3. Measurement of Biochemical Indicators

Creatinine (Cr), creatine kinase (CK), alkaline phosphatase (ALP), aspartate aminotransferase (AST), total protein (TP), albumin (ALB), globulin (GLB), total triglycerides (TG), total cholesterol (CHO), urea, alanine aminotransferase (ALT), lactate dehydrogenase (LDH), Ca^2+^, Mg, and P were measured using Lepu series blood biochemical reagents (Lepu, Lepu Medical Equipment Co., Ltd., Beijing, China) and an automated biochemical analyzer (HITACHI7020, Hitachi, Tokyo, Japan), according to the manufacturer’s instructions. Antioxidant indices, including total antioxidant capacity (T-AOC), malondialdehyde (MDA), superoxide dismutase (SOD), and glutathione peroxidase (GSH-Px), were measured using the Nanjing Jiancheng Colorimetric Test Kit (Nanjing Jiancheng Institute of Bioengineering, Nanjing, China).

### 2.4. Metabolomic Analysis of Serum and Data Processing

The serum was isolated by allowing the blood sample to stand for 30 min, followed by centrifugation at 3000 rpm for 10 min at 4 °C, and then immediately stored at −80 °C. Subsequently, 20 μL of serum was extracted with 120 μL of pre-cooled 50% methanol, vortexed for 60 s and stored at −20 °C. The supernatant was centrifuged at 4000 rpm for 20 min and transferred to a 96-well plate for LC-MS analysis. Additionally, 10 μL of the extraction mixture was reserved for quality control (QC) sample preparation. For LC/MS analysis, an UltiMate 3000 UPLC System (Thermo Fisher Scientific, Bremen, Germany) coupled with a Q-Exactive high-resolution tandem mass spectrometer (Thermo Fisher Scientific, Bremen, Germany) and a TripleTOF 6600 (SCIEX, Framingham, MA, USA) were utilized. The Q-TOF was operated in both positive and negative ion modes. The separations were conducted with an ACQUITY UPLC HSS T3 column (100 mm × 2.1 mm, 1.8-μm particle size, Waters, Milford, MA, USA) at 40 °C. The flow rate was 0.3 mL/min with a 2 μL injection volume, using a binary gradient elution of 0.1% formic acid in water (A) and 0.1% formic acid in acetonitrile (B). The gradient conditions were 0–0.8 min, 2% B; 0.8–2.8 min, 2% to 70% B; 2.8–5.6 min, 70% to 90% B; 5.6–6.4 min, 90% to 100% B; 6.4–8.0 min, 100% B; 8.0–8.1 min, 100% to 2% B; and 8.1–10 min, 2% B. The Q-TOF mass spectrometer parameters included a curtain gas pressure of 30 PSI, ion source gas 1 and 2 at 60 PSI each, and an interface heater temperature of 500 °C. The Ionspray voltage was set to 5000 V for positive and −4500 V for negative ion modes. Mass spectrometry data were acquired in IDA mode within a TOF mass range of 60 to 1200 Da, with mass accuracy calibration performed after every 20 samples. A pooled QC sample was analyzed every 10 sample runs to ensure system stability.

The mass spectrometry data were preprocessed using XCMS 3.5.1 software (Scripps Research, La Jolla, CA, USA), including peak extraction, grouping, retention time correction, a second round of grouping, and isotopic adduct annotation. After converting raw LC-MS files to mzXML format, data processing was performed in R using XCMS, CAMERA, and metaX. The normalized LC-MS data were analyzed using SIMCA-P 13.0 (Umetrics, Umea, Sweden) for multivariate analysis, including unsupervised principal component analysis (PCA) and supervised partial least squares discriminant analysis (PLS-DA) to examine metabolite changes between groups. Model evaluation was based on R2X and R2Y for explained X and Y variation and Q2 for predictive ability, with values above 0.5 indicating an acceptable model. Differential metabolites (DMs) were identified using PLS-DA variable importance in projection (VIP > 1), Student’s t-test (*p* < 0.05), and the fold-change (FC) values of each metabolite between the two groups were calculated. The DMs were further annotated using the KEGG database, and enrichment pathway analysis results were mapped.

### 2.5. DNA Extraction, 16S rDNA Sequencing, and Sequence Analysis

DNA was extracted from fecal samples using the CTAB method. The full-length 16S rRNA gene was amplified with primers 27F and 1492R, each barcoded. PCR was performed in a 20 μL reaction mixture, followed by gel electrophoresis to confirm the amplification. The PCR products were purified using the AxyPrep DNA Gel Extraction Kit and quantified with QuantiFluor^TM^-ST (Promega, Madison, WI, USA). SMRTbell libraries were prepared using the Pacific Biosciences SMRTbell^TM^ Template Prep Kit and sequenced on the PacBio RS II platform (Pacific Biosciences, Menlo Park, CA, USA). CCS reads were generated from subreads using SMRT Link, demultiplexed with Lima, and filtered for lengths between 1200 and 1650 bp. DADA2 was used for dereplication and chimera removal, generating feature tables and sequences. Diversity analyses (alpha and beta) were performed in QIIME2 (version 2018.11), and ASVs were annotated against the SILVA database (release 138). Visualizations were created in R (version 4.4.1).

### 2.6. Differential Metabolites Validation by UHPLC−MRM-MS Analysis

To verify the untargeted metabolomics data, we performed targeted analysis using an ultra-high-performance liquid chromatography-triple quadrupole mass spectrometer (UHPLC-QqQ-MS) in MRM mode, optimizing MRM parameters with standard solutions (Table S1), and finally quantified the selected metabolites. After thawing the serum samples, vortex for 10 s to mix. Take 400 μL of the sample and add 1500 μL of 20% acetonitrile-methanol internal standard solution; vortex for 3 min. Centrifuge at 12,000 r/min for 10 min at 4 °C, collect the supernatant, and transfer it to a centrifuge tube, then place it in a −20 °C freezer for 30 min. Subsequently, centrifuge again at 12,000 r/min for 3 min at 4 °C, place the supernatant in a vacuum centrifugal concentrator, and centrifuge at 1000 r/min and 30 °C for 3 h. Finally, add 300 μL of distilled water to the centrifuge tube, vortex for 30 s to mix, then filter for analysis. An Agilent 1290 Infinity UHPLC (Agilent Technologies, Santa Clara, CA, USA) coupled to a 6430 triple quadrupole (QqQ) mass spectrometer (Agilent Technologies, Santa Clara, CA, USA) (UHPLC-QqQ-MS) with an ESI source was used for performing qualitative analysis, with the specific experimental parameters being the same as in our previous experiments [11].

### 2.7. Statistical Analysis

Normality and homogeneity of variance tests were conducted on the data using SPSS 19.0 prior to performing independent sample t-tests. The Mann–Whitney non-parametric test was utilized for data that did not conform to a normal distribution. A *p*-value of less than 0.05 was regarded as statistically significant, while *p*-values ranging from 0.05 to 0.10 were considered indicative of tendencies. Additionally, Spearman’s correlation analysis was employed to assess the correlation between the relative abundance of microorganisms (genus) and the response intensity data of the corresponding metabolites.

## 3. Results

### 3.1. Differences in Heat Tolerance-Related Traits Between Hu Sheep and Xinggao Sheep

#### 3.1.1. Under Heat Stress Conditions Hu Sheep Exhibit More Stable Changes in Heat Tolerance Physiological Indicators Compared to Xinggao Sheep

**Figure 1:**
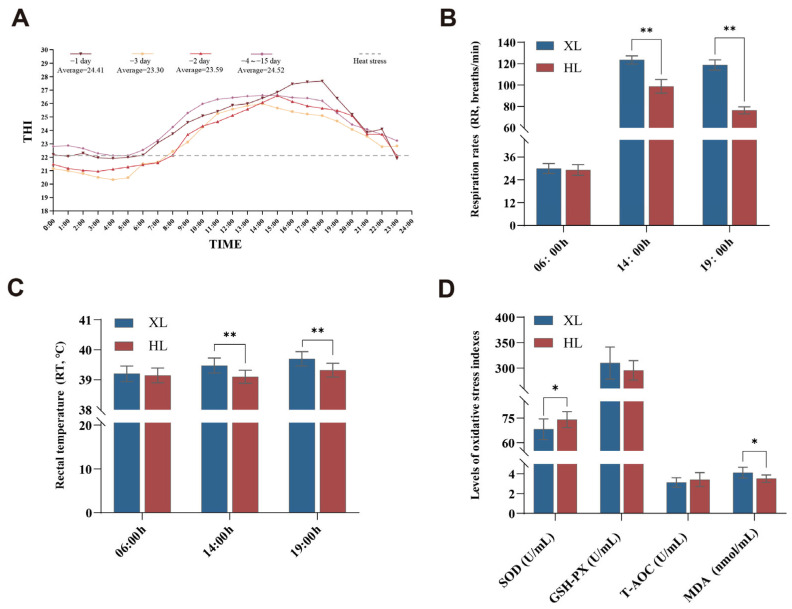
The weather trend before sampling and the measurement of physiological and antioxidant parameters in XL and HL groups during high-temperature conditions. (**A**) THI changes fifteen days prior to sampling, with −1 indicating the day before sampling, and so forth. Average represents the daily average THI, and the red dotted line marks the heat stress threshold; (**B**) respiration rates (RR); (**C**) rectal temperatures (RT); (**D**) levels of oxidative stress indexes; XL: Xinggao sheep; HL: Hu sheep; * *p* < 0.05, ** *p* < 0.01.

Traditionally, the Temperature–Humidity Index (THI) has served as a critical indicator for assessing sheep’s heat stress levels. The average THI calculated for the fifteen days preceding sampling indicates that the sheep were subjected to heat stress conditions (Figure 1A). Compared to the non-heat stress period, heat stress significantly reduced the average daily weight gain of the XL and HL groups, whereas the impact on the production performance of the HL group was relatively small, indicating stronger heat tolerance (Table S2).

Respiratory rate (RR) in both breeds increased with rising THI, and significant varietal differences in RR were observed at 14:00 and 19:00, with the Xinggao sheep (XL) exhibiting a significantly higher RR than the Hu sheep (HL). Moreover, the XL group demonstrated a greater rate of increase compared to the HL group (06:00 RR/14:00 RR; XL, FC = 4.12; HL, FC = 3.38) (Figure 1B). When THI decreased, the XL group showed a lower recovery rate (14:00 RR/19:00 RR; XL, FC = 0.04; HL, FC = 0.23). Rectal temperature (RT) measurements of sheep over three consecutive days in a high-temperature environment revealed that, like RR, RT also exhibited significant varietal differences at 14:00 and 19:00, with the XL group having a significantly higher RT than the HL group (Figure 1C). At both 14:00 and 19:00, the HT values in the XL group were significantly higher than those in the HL group (Table 1). The above results indicate that under heat stress conditions, as THI changes, the physiological parameters of Hu sheep are more stable compared to Xinggao sheep, suggesting that Hu sheep have stronger heat tolerance.

#### 3.1.2. Under Heat Stress Conditions Hu Sheep Exhibit Healthier Biochemical Parameters and Stronger Antioxidant Capacity Compared to Xinggao Sheep

As shown in Table 2, the levels of ALP in the XL group were significantly higher compared to those in the HL group, while Cr levels were significantly reduced in the XL group compared to the HL group (*p* < 0.05). The results of antioxidant indicators revealed that the superoxide dismutase (SOD) level in the HL group was significantly higher than that in the XL group, whereas malondialdehyde (MDA) level was significantly lower in the HL group compared to the XL group (*p* < 0.05) (Figure 1D).

### 3.2. Untargeted Metabolomic Analysis

**Figure 2:**
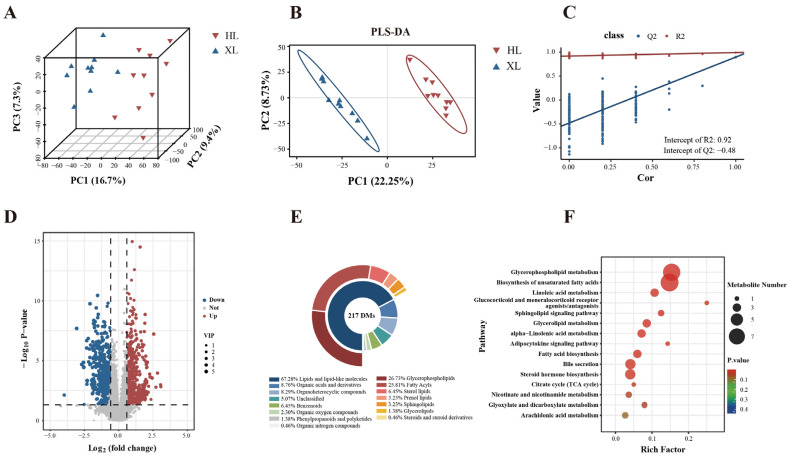
(**A**) Principal component analysis (PCA); (**B**) partial least squares discriminant analysis (PLS-DA); (**C**) PLS-DA permutation test plot; (**D**) volcano plot showing significant differences in metabolites between treatments selected by PLS-DA (VIP > 1, *p* < 0.05, FC = 1.5 or 1/1.5); (**E**) biochemical categories of identified differential metabolites are displayed in a pie chart; (**F**) enrichment pathways of differential serum metabolites in XL and HL groups under heat stress conditions.

In both positive and negative ion modes, 4709 and 7695 metabolite ion peaks were identified, respectively. Unsupervised principal component analysis (PCA) revealed a distinct separation between the XL and HL groups (Figure 2A), indicating significant metabolic differences and excellent intra-group repeatability under high-temperature conditions. A 7-fold cross-validated partial least squares discriminant analysis (PLS-DA) was performed to elucidate these differences further and identify potential biomarkers. The PLS-DA model displayed clear group separation with R²X = 0.992 and Q² = 0.897 (Figure 2B), confirming its credibility for interpreting the observed differences (Figure 2C). These results validate the reliability of both PCA and PLS-DA models.

Differential metabolites (DMs) were defined as those with a variable importance in projection (VIP) greater than 1, a *p*-value less than 0.05, and a fold change of ≥1.5 or ≤1/1.5. A total of 60 DMs were identified in positive ion mode and 157 in negative ion mode (Table S3). The volcano plot (Figure 2D) illustrated significant alterations in the HL group compared to the XL group, with 91 metabolites upregulated and 126 downregulated. These metabolites predominantly belong to lipids and lipid-like molecules (146), organic acids and derivatives (19), organoheterocyclic compounds (18), benzenoids (14), organic oxygen compounds (5), and others (15) (Figure 2E). Notably, several serum metabolite biomarkers—including cortisol (COR), dopamine (DA), 3-hydroxybutyric acid (BHBA), docosahexaenoic acid (DHA), eicosapentaenoic acid (EPA), L-carnitine (LC), acetyl-l-carnitine (ALC), and citric acid—are closely associated with heat sensitivity. Functional analysis using MetaboAnalyst revealed that the DMs are primarily involved in steroid hormone biosynthesis, glycerophospholipid metabolism, glycerolipid metabolism, bile secretion, and the biosynthesis of unsaturated fatty acids (Figure 2F).

Metabolites involved in steroid hormone biosynthesis—COR, 2-hydroxyestradiol, testosterone, and pregnenolone sulfate—exhibited significant changes. Specifically, COR and 2-hydroxyestradiol levels were significantly elevated in the XL group compared to the HL group (*p* < 0.01), while testosterone and pregnenolone sulfate decreased by approximately 0.50 to 0.60-fold (*p* < 0.01). Similarly, metabolites associated with the bile secretion pathway, including cholic acid, COR, and DA, increased by 1.81 to 3.44-fold in the XL group (*p* < 0.01), whereas LC decreased by 0.66-fold (*p* < 0.01).

In the biosynthesis of unsaturated fatty acids, key metabolites such as DHA, EPA, adrenic acid, stearic acid, palmitic acid, behenic acid, and lignoceric acid decreased by 0.41 to 0.64-fold in the XL group (*p* < 0.01), whereas linoleic acid increased by 1.96-fold (*p* < 0.01). Regarding glycerolipid metabolism, triglyceride 66:21, AcylGlcADG 60:6, and LysoPA 18:1 were significantly elevated in the XL group (1.53 to 3.33-fold increase, *p* < 0.01), while AcylGlcADG 66:18 (22:6/22:6/22:6), LysoPA 21:0, LysoPA 22:6, and LysoPA 22:0 decreased by 0.47 to 0.64-fold (*p* < 0.01). Similarly, metabolites involved in glycerophospholipid metabolism—PC (22:6/18:4), LysoPC 22:6, LysoPA 22:6, and PI (18:0/22:6)—decreased by 0.38 to 0.48-fold in the XL group (*p* < 0.01).

**Figure 3:**
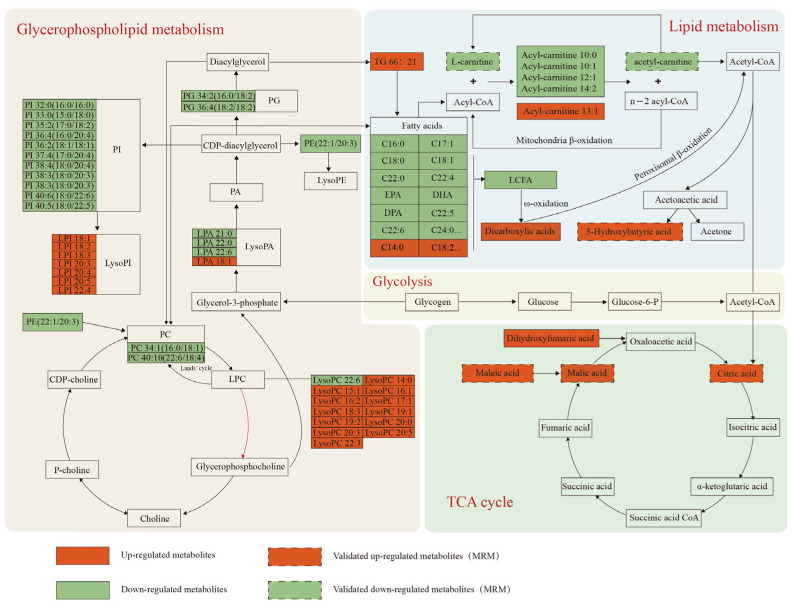
The network between different metabolites. Metabolites in brownish red and green represent higher or lower levels, respectively, in the XL group than in the HL group.

Notably, other metabolites closely related to the pathways above, such as BHBA, citric acid, dihydroxyfumaric acid, and maleic acid, significantly increased in the XL group (1.3 to 2.1-fold, *p* < 0.01), whereas ALC decreased by 0.53-fold (*p* < 0.01). These metabolites were integrated and represented as a network graph to illustrate their interrelationships (Figure 3).

### 3.3. Microbial Community Composition and Diversity Analysis

**Figure 4:**
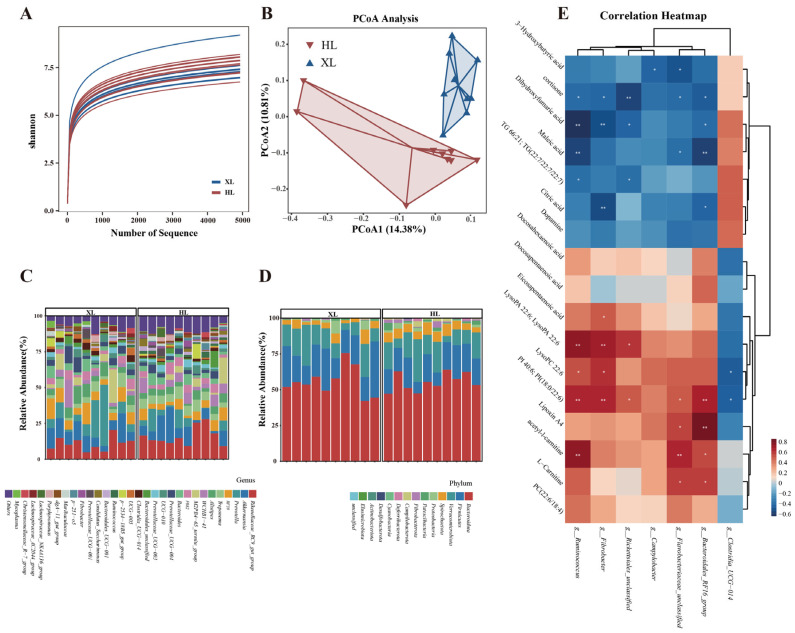
(**A**) Differential analysis of gut microbiota in the XL group and HL group under heat stress conditions. rarefaction of the different samples; (**B**) differences in principal coordinate analysis (PCoA) of intestinal microbiome XL and HL groups. The red dots represent the samples of the XL group, and the blue dots represent the samples of the HL group. The distance between the two points represents the difference in intestinal microbiota; (**C**) the taxonomic distribution between XL group and HL group samples (each color represents the relative abundance of a taxonomic bacterium). Between-group at the phylum level (top 14); (**D**) between-group at the genus level (top 30); (**E**) heatmap of correlations between gut microbiota and differential metabolites. Red indicates a positive correlation, blue indicates a negative correlation; * *p* < 0.05, ** *p* < 0.01.

The microbial community composition was analyzed using 16S rDNA gene sequencing, yielding 942,829 clean reads from 20 samples, with an average of 47,141 reads per sample (Table S4). Rarefaction curves plateaued beyond 1000 sequences, indicating sufficient sequencing depth (Figure 4A). Alpha diversity indices (Shannon, Chao1, Simpson) showed no significant differences between the XL and HL groups (*p* > 0.05, Table 3). However, beta diversity analysis revealed distinct microbial compositions between the groups. PCoA plots demonstrated clear separation between HL and XL groups (Figure 4B), and ANOSIM confirmed significant differences in bacterial communities (R = 0.19, *p* = 0.005, Figure S1). These results suggest notable distinctions in microbial community structures despite some common characteristics under heat stress conditions.

To investigate the microbial community structure of the XL and HL groups, we analyzed their taxonomic composition at both the phylum and genus levels. As shown in Figure 4C, a total of 14 phyla were identified within the XL and HL groups. The dominant phyla, exhibiting an average relative abundance ≥ 10%, included *Bacteroidota* (55.7% in XL and 55.4% in HL), *Firmicutes* (21.8% in XL and 17.3% in HL), and *Verrucomicrobiota* (14.7% in XL and 15.1% in HL). Furthermore, we conducted a detailed analysis of the top 30 genera present in these samples. *Rikenellaceae_RC9_gut_group* (10.9%), *Akkermansia* (10.6%), and *Prevotella* (11.4%) were the dominant genera in the XL group. In the HL group, the dominant genera were *Rikenellaceae_RC9_gut_group* (15.9%) (Figure 4D).

To further elucidate the differences in gut microbiota between the XL and HL groups under heat stress conditions, we employed Wilcox’s t-test to identify differential microorganisms. Significant differences were observed in the abundances of *Fibrobacterota* and *Campilobacterota* at the phylum level between the two groups. Specifically, the XL group exhibited a notable reduction in the abundance of both *Fibrobacterota* and *Campilobacterota* compared to the HL group. At the genus level, *Ruminococcus*, *Bacteroidales_RF16_group*, *Fibrobacter*, *Rickettsiales_unclassified*, *Flavobacteriaceae_unclassified*, and *Campylobacter* were found to be significantly higher in the HL group than in the XL group. At the same time, *Clostridia_UCG-014* was significantly reduced in the HL group.

### 3.4. Targeted Qualitative Validation of Differential Metabolites

**Figure 5:**
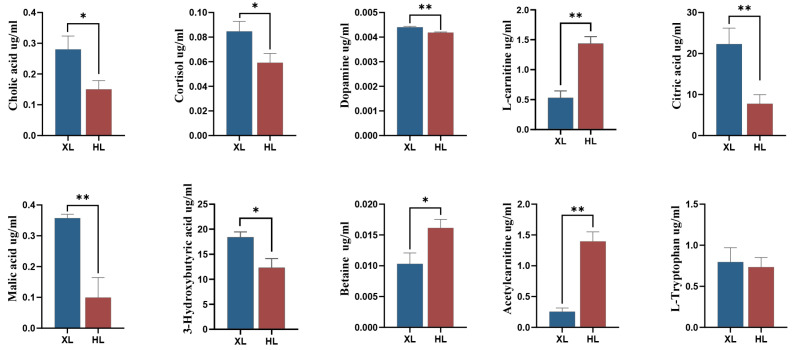
Verification of specific metabolite concentrations by UHPLC-MRM-MS; * *p* < 0.05, ** *p* < 0.01.

The UHPLC-MRM-MS analysis results confirmed that in the bile secretion pathway, the levels of bile acids, COR, and DA in the XL group were significantly higher compared to the HL group, and the LC level was also significantly reduced. Metabolites related to the tricarboxylic acid cycle, such as citric, malic, and maleic acids, were also significantly elevated. Other metabolites related to heat stress, such as 3-hydroxybutyrate, were significantly increased, while betaine and acylcarnitines were significantly decreased. Although we observed a significant change in the tryptophan pathway in the non-targeted metabolomics data, no significant change in the level of L-tryptophan was found in the quantitative detection (Figure 5).

### 3.5. Correlations Between Microbial Communities and Metabolites

The correlation heatmap indicates that dihydroxyfumaric acid negatively correlates with *Ruminococcus*, *Fibrobacter*, *Rickettsiales_unclassified*, and *Bacteroidales_RF16_group*. Additionally, maleic acid is negatively correlated with *Ruminococcus*, *Bacteroidales_RF16_group*, and *Flavobacteriaceae_unclassified*. Conversely, lipoxin A4 positively correlates with the *Bacteroidales_RF16_group* and *Flavobacteriaceae_unclassified* (Figure 4E). Detailed correlations between other metabolites and microorganisms can be found in Table S5.

## 4. Discussion

### 4.1. Differences in Heat Tolerance-Related Traits

We found that, compared to the non-heat stress period, heat stress significantly reduced the average daily weight gain of the two sheep breeds (XL and HL groups) by −77% and −63%, respectively. This suggests that heat stress has a lesser impact on the production performance of the HL group, indicating a stronger adaptability. To further explore the molecular mechanisms underlying this phenomenon, we used physiological and biochemical indicators as phenotypes and employed metabolomics and gut microbiomics to compare the adaptive differences between the two sheep breeds under heat stress conditions. Our results will contribute to providing a theoretical basis and potential molecular markers for the breeding of heat-resistant sheep.

#### 4.1.1. Differences in Physiological Indicators of Heat Stress Conditions

In environments with high temperatures, animals first manage heat production and heat release via physiological responses like body temperature, heart rate, and respiratory rate to maintain homeostasis under heat stress conditions. Evaporative cooling, the primary mechanism for thermoregulation in high temperatures, is chiefly achieved through sweating and respiratory evaporation. Unlike other ruminants, sheep predominantly rely on an increased respiratory rate to enhance evaporative cooling, which accounts for 60–90% of total heat dissipation, while sweating contributes only about 10% to this process [12]. Consequently, respiratory rate has emerged as a crucial physiological indicator of heat tolerance in sheep.

In this study, we observed that respiratory rate (RR) is affected by breed and time. As the THI increased from 06:00 to 14:00, the RR of both breeds increased; however, the increase in the XL group (FC = 4.12) was significantly greater than that in the HL group (FC = 3.38). Furthermore, as THI decreased from 14:00 to 19:00, the decline in RR for the XL group (FC = 0.04) was slower than that in the HL group (FC = 0.23). These findings indicate that the RR of the XL group is more sensitive to fluctuations in THI and recovers more slowly. Rectal temperature (RT) is typically employed to assess the thermoregulatory capacity of animals under heat stress conditions. Our research revealed that, like RR, the RT of the XL group was significantly higher than that of the HL group at both 14:00 and 19:00. This suggests that the XL group may mitigate heat absorption and enhance heat dissipation by increasing body temperature, indicating a greater sensitivity to high-temperature environments. Furthermore, the HT values (heat tolerance index) were consistent with the abovementioned results. These results indicate that the XL group is more sensitive to high-temperature environments.

#### 4.1.2. Differences in Biochemical Indicators of Heat Stress Conditions

Heat-induced oxidative stress leads to excessive ROS like superoxide anion (O2−), nitric oxide (NO), and hydroxyl radicals (HO·), damaging cell membranes and mitochondrial activity. Superoxide dismutase (SOD) is a crucial enzyme found in various tissues that protects cells from damage caused by superoxide anion radicals and their reactive products; its levels serve as an indicator of the body’s capacity to scavenge free radicals. Malondialdehyde (MDA), a byproduct of lipid peroxidation, reflects the degree of free radical generation within the body [9]. In our study, we observed that the SOD levels in the HL group were significantly higher than those in the XL group, while MDA levels exhibited a decreasing trend in the HL group compared to the XL group (*p* < 0.05). These findings align with Liao et al. [4], who found that heat-tolerant Xuanhan cattle exhibited higher SOD and lower MDA levels among three beef cattle breeds. Our results suggest that the HL group has less oxidative stress and a more robust antioxidant capacity under heat stress conditions than the XL group.

Creatinine (Cr), a byproduct of muscle metabolism from creatine and creatine phosphate, is crucial for energy metabolism in skeletal and cardiac muscles. Its levels correlate with muscle mass and activity, making serum Cr a marker for muscle activity and mass [13]. Furthermore, low Cr levels have been associated with inadequate nitrogen intake [14]. Our results indicate that the Cr levels of serum in both the XL and HL groups were below the normal range [15]. We hypothesize that this may be attributed to decreased muscle activity, characterized by increased periods of lying down, and reduced nitrogen intake due to decreased feed intake in sheep exposed to high heat conditions. Similar findings have been reported by Li et al. [16]. The significantly lower serum Cr levels in the XL group compared to the HL group may indicate lower muscle activity and protein intake. It is well established that alkaline phosphatase (ALP) serves as a key biomarker for liver injury, with elevated levels often associated with alterations in hepatic metabolic function. The liver, being the primary metabolic organ in mammals, plays a crucial role in lipid metabolism. Numerous studies have demonstrated that oxidative stress leads to liver damage and disrupts lipid metabolism by promoting the production of ROS and inhibiting the antioxidant system [17,18]. Therefore, we hypothesize that the observed decrease in antioxidant levels, the increase in ALP levels, and the abnormalities in lipid metabolism identified in the serum metabolomics of the XL group may be closely related to the liver’s metabolic state.

### 4.2. Metabolic Differences in Heat Stress Conditions

Environmental stress factors lead to variations in energy metabolism and metabolite levels among different sheep breeds through gene regulatory processes. When exposed to heat stress, the metabolic differences between the two sheep breeds are predominantly observed in pathways related to steroid hormone biosynthesis, glycerophospholipid metabolism, glyceride metabolism, bile secretion, and unsaturated fatty acid biosynthesis pathways. Our research findings revealed these metabolic pattern differences under heat stress, providing a theoretical basis for regional breeding and management strategies.

#### 4.2.1. Stress-Related Metabolite Changes

The hypothalamic–pituitary–adrenal (HPA) axis and the sympathetic-adrenal-medullary (SAM) axis are vital systems that regulate homeostasis in animals exposed to stress stimuli. Upon activation by elevated heat conditions, these axes produce adrenocortical hormones (such as cortisol) and adrenal medullary hormones (such as catecholamines) [19]. Cortisol (COR), the principal stress hormone in ruminants, is synthesized in greater quantities during stress as an adaptive response. This increase triggers hepatic gluconeogenesis and suppresses the immune system [20]. COR acts as a metabolic breakdown signal, generally enhancing the mobilization of body fat; the newly produced free fatty acids (FFA) subsequently provide energy through *β*-oxidation to meet heightened energy demands [21]. Our study observed a significant increase in serum COR levels in the XL group compared to the HL group. Research by Tejawsi et al. [22] indicates that different cattle breeds exhibit varying responses to heat stress, with low-heat-tolerant hybrid cattle showing elevated serum COR levels compared to local breeds. Additionally, Archana et al. [23] also found that during summer, Osmanabadi goats exhibited significantly higher serum COR concentrations than heat-tolerant Salem Black goats, whose serum COR levels fluctuated more stably with temperature changes. Furthermore, other studies have demonstrated that elevated COR levels can diminish the tolerance to high temperatures [24]. Collectively, these studies suggest that breeds more sensitive to heat generally have higher serum COR concentrations.

Dopamine (DA), a precursor to adrenaline and noradrenaline, increases in response to heightened stress and environmental discomfort. Under stress conditions, circulating DA is mainly produced by the sympathetic nerves and the adrenal medulla [25]. Research has demonstrated that increased heat stress in mice stimulates the hypothalamus, resulting in elevated serum DA levels, which are associated with physiological distress, including increased heart rate and arterial pressure, as well as hypothalamic distortions during severe heat exposure [26]. In our study, we observed that the serum DA levels in the XL group were significantly higher than those in the HL group. This finding aligns with the work of Burdina et al. [27], who identified a negative correlation between heat tolerance and DA levels in fruit flies. Our results indicate that, at the same environmental temperature, the serum levels of COR and DA in the XL group are substantially elevated compared to those in the HL group, suggesting that the XL group exhibits greater sensitivity to heat stress. These alterations may indicate that the HPA axis and SAM axis in the XL group experience more pronounced disturbances under high-temperature conditions.

#### 4.2.2. Lipid Metabolism Changes

Mammals typically store excess energy in the form of triglycerides (TG), which are mobilized to meet energy demands during periods of energy deficiency in the body [28]. Under the regulation of the HPA axis, COR and catecholamine hormones (DA, adrenaline, and noradrenaline) serve as signals for catabolic metabolism; during heat stress, these hormones stimulate fat mobilization, resulting in increased circulating levels of TG [21,29]. Research conducted by Swanson et al. [29] demonstrated that lambs exposed to heat stress exhibited elevated circulating TG levels compared to their pair-fed counterparts, attributed to heightened catecholamine levels in the bloodstream. In a study in Holstein-Friesian crossbred cattle (which are crossbred with local cattle breeds known for their superior adaptability), Ihsanullah and colleagues observed that individuals with a higher proportion of Holstein-Friesian genetics were more sensitive to heat and displayed enhanced fat mobilization, as indicated by elevated circulating TG levels, consistent with the findings of this study [30]. Our research indicates that the XL group has higher serum TG levels (Figure 3), suggesting more intense lipid mobilization in the XL group, potentially indicative of higher heat sensitivity. Furthermore, research by Bai and Anna has established that cortisol, through the activation of glucocorticoid receptors, upregulates the expression of the fatty acid translocase CD36, leading to increased TG levels in circulation and the liver. Additionally, elevated plasma cortisol levels also enhance the expression of β-oxidation genes such as ApoB and MTTP, which may further elucidate the more pronounced lipid mobilization observed in the XL group [31,32].

Fatty acid oxidation (FAO) encompasses the generation of free fatty acids through lipolysis during periods of starvation and metabolic stress, followed by their β-oxidation in mitochondria or peroxisomes to produce energy. Mitochondrial β-oxidation serves as the primary pathway for FAO, where free fatty acids are transported via carnitine to produce acetyl-CoA, a substrate for ketone body synthesis and an essential fuel source for the TCA cycle [33]. Therefore, FAO plays a critical role in maintaining energy homeostasis. However, under specific physiological or pathological conditions, peroxisomal β-oxidation becomes increasingly significant, particularly during heightened lipid flux, fasting, or mitochondrial FAO defects. During these times, long-chain fatty acids initially undergo ω-oxidation in peroxisomes, resulting in the production of dicarboxylic acids (DCAs), which are then shortened via β-oxidation to generate DCAs and acetyl-CoA, thereby supplying energy to the body [34]. Chen et al. [35] reported that heat stress can lead to elevated levels of DCAs in cold-water fish, activating a secondary oxidation pathway (peroxisomal oxidation). This finding aligns with our research outcomes. Our study shows that the XL group exhibited increased levels of DCAs and decreased levels of acetyl-l-carnitine (ALC) and L-carnitine (Figure 3), suggesting that mitochondrial β-oxidation may be inhibited under heat stress in the XL group. The elevated levels of DCAs indicate that peroxisomal ω-oxidation may be activated, potentially indicating a competitive mechanism between peroxisomal β-oxidation and mitochondrial β-oxidation, which requires further investigation.

The ALC is the predominant acyl-carnitine found in plasma and plays a crucial role in metabolism, including regulating glucose homeostasis, stimulating glycogen synthesis, and increasing ATP concentration in plasma [36]. When fatty acids are activated to form acyl-CoA and undergo multiple cycles of β-oxidation, they produce several acetyl-CoA molecules. In conditions where pyruvate precursors (e.g., glucose) are abundant, these acetyl-CoA molecules accumulate as ALC; conversely, when such precursors are scarce, the acetyl groups are released for metabolic utilization [37]. Thus, the observed decrease in ALC levels in the serum of the XL group may suggest an increased utilization of ALC to regulate the energy status of this group.

Hydroxybutyric acid (BHBA), one of the primary ketone bodies, serves as a direct marker of the energy status in mammals. Its excessive accumulation indicates enhanced fat mobilization and insufficient dietary energy supply [38]. Research indicates that the rise in BHBA levels in animals under heat stress conditions is attributable to the combined effects of hormonal regulation and reduced feed intake [39]. During this process, fatty acids derived from fat are released from TG through lipolysis, and after undergoing β-oxidation, they produce acetyl-CoA, which further condenses into ketone bodies. These ketone bodies are then transported to the TCA cycle for utilization [40]. Our research demonstrated a significant increase in serum BHBA levels in the XL group compared to the HL group (Figure 3). He and his colleagues found that heat stress leads to increased BHBA levels in milk, serum, and liver, with BHBA in these samples exhibiting good sensitivity and specificity for diagnosing heat stress, thereby serving as a potential biomarker for heat stress in dairy cows [39,41,42]. The increase in BHBA levels results from heightened fat mobilization in the body; however, when other tissues reach their saturation point in utilizing ketone bodies, and not all can be oxidized by the citric acid cycle, this leads to a substantial accumulation of BHBA in circulation [43]. Therefore, the elevated levels of TG and BHBA indicate more intense fat mobilization in the XL group.

#### 4.2.3. TCA Cycle-Related Metabolic Changes

Heat stress is known to accelerate the TCA cycle, resulting in an accumulation of TCA intermediates in the plasma [4]. In this study, we observed that levels of citric acid, malic acid, maleic acid, and dihydroxyfumaric acid in the serum were significantly higher in the XL group compared to the HL group (Figure 3). This finding suggests that during the heat stress period, the demand for ATP in Xinggao sheep increased, thereby stimulating a rise in the levels of TCA cycle intermediates. This phenomenon may be attributed to enhanced β-oxidation of fatty acids in the XL group, leading to elevated levels of acetyl-CoA, indicating increased energy demand during this time.

#### 4.2.4. Glycerophospholipid Metabolism Changes

Phosphatidylcholine (PC) and phosphatidylethanolamine (PE) are the predominant phospholipids in plasma, accounting for approximately 76% and 17% of the total, respectively, with PC serving as a major component of the cellular phospholipid bilayer. The composition of fatty acyl chains in PC influences the biophysical properties of membranes [44]. ω-3 fatty acids primarily exert their biological functions by binding to glycerophospholipids within cell membranes. For example, cell membranes rich in DHA exhibit higher permeability, facilitating the rapid regulation of cell functions by small molecules. Additionally, ω-3 fatty acids display their antioxidant properties by elevating intracellular levels of glutathione [45,46]. Our study found significant decreases in DHA, EPA, PC (22:6/18:4), LPC (22:6), LPA (22:6), and PI (22:6) in the XL group (Figure 3). This suggests that the downregulation of the glycerophospholipid metabolism pathway is attributable to a reduction in de novo synthesized fatty acids rather than alterations caused by phospholipase activity. Research by Chauhan et al. [47] indicates that sheep subjected to heat stress exhibit reductions in muscle tissue levels of EPA and DHA compared to those under antioxidant protection in similar conditions, demonstrating the impact of oxidative stress on ω-3 PUFA levels in sheep. Li et al. [48] also reported analogous findings in their research. Consequently, the observed reduction in ω-3 fatty acids and glycerophospholipids may be associated with the lower antioxidant capacity in the XL group. This observation further confirms that the oxidative stress induced by high temperature has a more significant impact on the XL group.

### 4.3. Differences in Gut Microbiota Under Heat Stress Conditions

In ruminants, *Ruminococcus* plays a crucial role in the degradation and fermentation of plant cellulose. It is typically abundant under healthy conditions but is significantly reduced during disease [49,50]. A study by Du et al. [51] on strategies to alleviate heat stress in cattle found that an increase in *Ruminococcus* abundance may improve the antioxidant capacity of cattle under heat stress by regulating the pentose phosphate pathway to produce NADPH. Saad et al. [50] demonstrated that supplementing with *Ruminococcus* sp. during heat stress significantly reduced the expression of heat stress-related genes (Bax, NF-κβ1, HSP 70) in the bursa of Fabricius and enhanced antioxidant capacity. Our research findings indicate that the relative abundance of *Ruminococcus* at the genus level in the feces of the XL group is significantly lower than that observed in the HL group. This discrepancy may suggest that the high-temperature environment exerts a more pronounced effect on the cellulose degradation capacity of the XL group. Moreover, the reduction in *Ruminococcus* abundance may be associated with the lower antioxidant capacity of the XL group.

In this study, we observed a negative correlation between the low abundance of *Ruminococcus* in the XL group and an upregulation of dihydroxyfumaric and maleic acids. This outcome may be related to *Ruminococcus* being a major butyrate-producing bacterium, with butyrate playing a key role in energy supply and fat synthesis in ruminants [49]. The reduction in *Ruminococcus* quantity leads to decreased butyrate supply, which may further activate the glyoxylate and dicarboxylate metabolic pathways (upregulation of dihydroxyfumaric acid). This metabolic shift prompts the organism to utilize fatty acids as a carbon alternative source to maintain energy levels, as evidenced by the increased levels of maleic acid and dihydroxyfumaric acid within the glyoxylate, dicarboxylate, and butanoate metabolism pathways. Similarly, *Fibrobacter*, which possesses a robust capacity for cellulose degradation, also exhibited a decline in abundance within the XL group. This reduction further suggests that its cellulose degradation capacity diminishes under high-temperature conditions compared to the HL group.

*Bacteroidales* play a crucial role in maintaining gut microbiota balance in a healthy state. They are instrumental in degrading polysaccharides to produce short-chain fatty acids and by producing capsular polysaccharides that enhance the antigenicity of the intestinal surface, thereby improving the host’s immune capacity [52]. Guo et al. [53] reported that the accumulation of *Bacteroidales* in the colons of Qinghai donkeys helps them adapt to high-altitude climates and the complex changes in their internal environment. This finding aligns with our results, where we observed a significantly higher abundance of *g__Bacteroidales_RF16_group* in the more heat-adaptive HL group compared to the XL group. Furthermore, He et al. [54] found that the significant reduction in *Bacteroidales* abundance in the intestines of pregnant sows under heat stress is a key factor leading to decreased levels of short-chain fatty acids. This reduction not only disrupts intestinal homeostasis but also triggers inflammatory responses. It is worth noting that short-chain fatty acids play a particularly critical role in energy metabolism for ruminants compared to other animals. Therefore, we speculate that the differences in heat tolerance between the XL and HL groups may be closely linked to the involvement of *Bacteroidales* in fatty acid metabolism. Additionally, prior studies have shown that an increased *Firmicutes*-to-*Bacteroidota* ratio can enhance energy utilization efficiency in animals [55]. Interestingly, research on pregnant sows in heat stress environments has found that when heat stress induces a negative energy balance, the *Firmicutes*-to-*Bacteroidota* ratio in the gut microbiota increases as an adaptive response [56]. This is consistent with our findings, where the *Firmicutes*-to-*Bacteroidota* ratio in the XL group was higher than that in the HL group (*p* = 0.07). This observation aligns with the negative energy balance state reflected in the metabolomics data of the XL group. Therefore, we hypothesize that the increased *Firmicutes*-to-*Bacteroidota* ratio may represent an adaptive mechanism for regulating energy metabolism under the heat stress in the XL group. However, further investigation is required to validate this hypothesis.

Our results indicate that the genus *g__Bacteroidales_RF16*_group is positively correlated with the downregulated metabolite Lipoxin A4 in the XL group, exhibiting the highest correlation (RHO = 0.78). Studies have shown that *Bacteroidales* promote intestinal epithelial barrier function by regulating IL-6 secretion, which protects the intestinal barrier and enhances the host’s resistance to intestinal pathogens such as Citrobacter rodentium [57]. Lipoxin A4, derived from arachidonic acid, possesses cytoprotective and anti-inflammatory properties. Furthermore, Lipoxin A4 and its analogs can alleviate colitis, reduce adipose tissue inflammation, and regulate the secretion of chemokines (IL-6, IL-8) [58,59]. Our findings suggest that, at the genus level, the abundance of *g__Bacteroidales_RF16*_group in the feces of the HL group is higher compared to the XL group. This information may indicate that under high-temperature conditions, the HL group may enhance adaptability and maintain gut microbiota balance and immune function through a higher abundance of *g__Bacteroidales_RF16*_group.

## 5. Conclusions

In summary, under heat stress conditions, Hu sheep exhibit greater heat tolerance compared to Xinggao sheep. Specifically, the respiratory rate and rectal temperature of Hu sheep show more stable fluctuations with THI values, indicating a stronger thermoregulation capacity. Additionally, Hu sheep demonstrate superior antioxidant capacity and superior biochemical indicators, further supporting their enhanced heat tolerance. Metabolomics analysis reveals that Xinggao sheep tend to synthesize higher levels of cortisol and dopamine in response to heat stress. This response enhances fat mobilization and leads to the accumulation of tricarboxylic acid cycle metabolites, which may represent one of their metabolic strategies for coping with high temperatures. In contrast, Hu sheep possess higher abundances of Ruminococcus, Fibrobacter, and Bacteroidales_RF16_group in their gut microbiota, indicating a stronger cellulose degradation ability and a more advantageous gut microbial composition. In conclusion, this study combines physiological and biochemical indicators with serum metabolomics and gut microbiology to reveal the differences in heat tolerance mechanisms between the two breeds. These findings provide a theoretical basis for improving the management of these breeds under heat stress conditions and developing more effective breeding strategies.

## Figures and Tables

**Table 1:** Heat resistance indices of HL and XL groups at different time periods.

	Heat Tolerance Index (HT)		
Time	XL (MEAN ± SD)	HL (MEAN ± SD)	*p*-Value
06:00	2.00 ± 0.09	2.00 ± 0.10	0.978
14:00	5.13 ± 0.13	4.38 ± 0.22	<0.01
19:00	4.97 ± 0.16	3.62 ± 0.11	<0.01

*p*-values were calculated from Student’s *t*-test with a threshold of 0.05; MEAN ± SD, mean ± standard deviation; XL: Xinggao sheep, HL: Hu sheep.

**Table 2:** Comparison of differences in serum biochemical indices between XL and HL groups under heat stress environment.

	Changes in the Concentration or Activity of Biochemical Parameters		
Items	XL (MEAN ± SD)	HL (MEAN ± SD)	*p*-Value
UN (mmol/L)	1.57 ± 0.22	1.41 ± 0.17	0.11
LDH (U/L)	342.82 ± 82.97	318.77 ± 83.30	0.53
ALT (U/L)	18.41 ± 10.50	20.03 ± 7.75	0.70
AST (U/L)	92.71 ± 48.92	96.49 ± 13.09	0.41
ALP (U/L)	177.83 ± 59.76	104.68 ± 50.91	<0.01
TP (g/L)	59.34 ± 15.12	67.49 ± 14.25	0.13
ALB (g/L)	21.94 ± 3.40	23.22 ± 3.57	0.42
CHOL (mmol/L)	1.52 ± 0.41	1.62 ± 0.51	0.64
Ca^2+^ (mmol/L)	2.13 ± 0.33	2.25 ± 0.25	0.57
Mg (mmol/L)	0.98 ± 0.21	1.12 ± 0.13	0.10
P (mmoI/L)	1.46 ± 0.38	1.61 ± 0.33	0.34
Cr (μmol/L)	30.65 ± 4.73	38.37 ± 6.40	<0.05
CK (U/L)	82.69 ± 61.81	71.93 ± 32.91	0.88
GLB (g/L)	37.40 ± 12.38	44.27 ± 10.97	0.13

UN: urea nitrogen, LDH: lactate dehydrogenase, ALT: alanine aminotransferase, AST: aspartate aminotransferase, ALP: alkaline phosphatase, TP: total protein, ALB: albumin, CHOL: cholesterol, Ca^2+^: calcium ion, Mg: magnesium, P: phosphorus, Cr: creatinine, CK: creatine kinase, GLB: globulin. The results are presented as means; data were presented as means ± SD; XL: Xinggao sheep, HL: Hu sheep.

**Table 3:** Intestinal microbial diversity of XL and HL group.

Sample	DG (MEAN ± SD)	XH (MEAN ± SD)	*p*-Value
observed_otus	204.0 ± 115.61	201.4 ± 52.19	0.38
shannon	7.02 ± 0.60	7.10 ± 0.44	0.38
simpson	0.99 ± 0.00	0.99 ± 0.00	0.35
chao1	206.21 ± 121.36	202.16 ± 52.85	0.38
goods_coverage	1.0 ± 0.00	1.0 ± 0.00	0.82
Shannon’s evenness	0.93 ± 0.01	0.93 ± 0.01	0.55

*p*-values were calculated from Student’s t-test with a threshold of 0.05; data were presented as means ± SD; XL: Xinggao sheep, HL: Hu sheep.

## Data Availability

The original contributions presented in this study are included in the article/Supplementary Material. Further inquiries can be directed to the corresponding authors.

## Supplementary Materials

The following supporting information can be downloaded at https://www.mdpi.com/article/10.3390/metabo15010040/s1, Figure S1: ANOSIM analysis; Table S1: Optimised detection conditions for mass spectrometry; Table S2: Average daily gain; Table S3: Non-targeted metabolomics analysis and screening results; Table S4: Sample Tags Total table of distribution; Table S5: Correlation between metabolites and microorganisms.
