# Supplementary material for: Heat Tolerance Differences Between Hu Sheep and Hu Crossbred Sheep in Microbial Community Structure and Metabolism
Source: Metabolites. 2025 Jan 10;15(1):40. doi: 10.3390/metabo15010040 (PMC11768064; doi:10.3390/metabo15010040)
Supplement: Supplementary file 1 [file metabolites-15-00040-s001.zip › Table S1 S2 S4.pdf]

Table S1 Optimised detection conditions for mass spectrometry

Metabolite	Precursor lon	Product lon	Fragmentor	Collision Energy	*rt(s)*	ESI ionisation model	Cell Accelerator Voltage
3-Hidroxybutyric acid	103	59.1	60	12	1.283	P	4
Maleic acid	115	71.1	55	12	0.933	N	4
Malic acid	133	115	60	12	0.675	N	4
Dopamine	152.1	122.1	100	18	1.013	N	4
Betaine	162.1	45	40	2	0.56	N	4
Citric acid	191	111	60	8	1.032	N	4
L-Tryptophon	203.1	116.1	100	12	5.645	N	4
ACs	204.1	85.1	60	12	0.834	P	4
L-CARNITINE	206.1	45	60	12	0.548	N	4
COR	363.2	363.2	120	1	5.816	N	4

Table S2 Average daily gain

Group	XL	HL
TN (kg)	0.11±0.03	0.08±0.02
HS (kg)	0.02±0.03	0.03±0.02
Decreased Percentage	-77%	-63%

Table S4.Sample Tags Total table of distribution

Sample	Clean tags
R0DG01	51641
R0DG02	35254
R0DG03	45777
R0DG04	54235
R0DG05	46673
R0DG06	38556
R0DG07	46841
R0DG08	36449
R0DG09	47523
R0DG10	58714
R0XH01	57632
R0XH02	40233
R0XH03	54475
R0XH04	44624
R0XH05	42740
R0XH06	38519
R0XH07	47555

R0XH08	51232
R0XH09	51320
R0XH10	52836
