# Supplementary figures and images for: Heat Tolerance Differences Between Hu Sheep and Hu Crossbred Sheep in Microbial Community Structure and Metabolism
Source: Metabolites. 2025 Jan 10;15(1):40. doi: 10.3390/metabo15010040 (PMC11768064; doi:10.3390/metabo15010040)

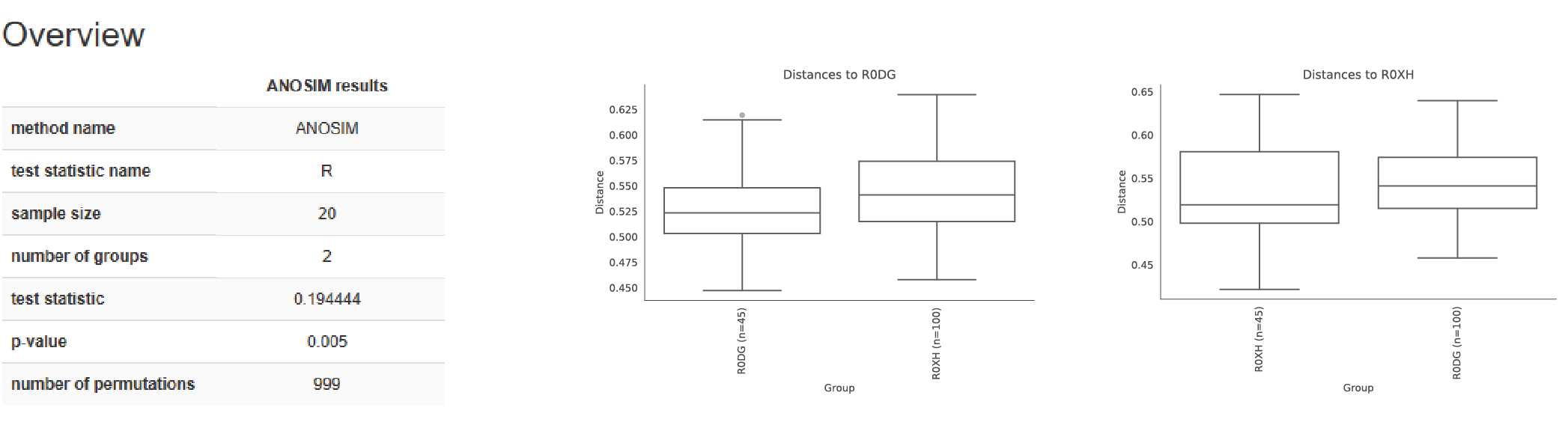

Supplement: Supplementary file 1 [file metabolites-15-00040-s001.zip › Figure S1 ANOSIM analysis.tif]
